# Book Notices

**Published:** 1875-02

**Authors:** 


### *Book Notices.*

Eating for Strength. A Book comprising: 1. The science of eating. 2. Receipts for wholesome cooking. 3. Receipts for wholesome drinks. 4. Answers to ever-recurring questions. By M. L. Holbrook, M. D., Editor "*Herald of Health*," etc., etc. New York. Wood & Holbrook, 13 and 15 Laight Street. 1875.

We have examined this work with some care, and find many good suggestions and valuable receipts for the better preparation of food. Such works should command the attention of the profession, and through them the great public, on so important a theme.

Transactions of the Texas State Medical Association. Sixth Annual Session, 1874. Held in Dallas, April 7th, 8th, 9th and 10th.

These transactions give evidence of growth and scientific strength among the medical men of the "Lone Star State." The volume is well filled with papers, many of which are too valuable to be embalmed in the archives of the Association and buried from the medical world. We suggest to our friends everywhere, when assembled in local or State Societies, the importance of having papers of more than ordinary excellence referred to a committee, and then given to the profession if deemed worthy.

TRANSACTIONS OF THE MEDICAL SOCIETY OF THE DISTRICT OF COLUMBIA: October, 1874. January, 1875.

Now here is a new idea—the publication of quarterly transactions. The pamphlets before us are meritorious.

Scleritis Syphilitica: Its pathology, course and treatment. By Fred. R. Sturgis, M. D.

Report of the Committee on Sick, Hospital and Sanitary Measures. Chicago Relief and Aid Society.

A Retrospect of the Struggles and Triumph of Ovariotomy in Philadelphia, with some additional remarks on allied subjects. The Annual Address before the Philadelphia County Medical Society. By Washington M. Atlee, M.D.

Report of One Hundred and Five Cases of Operations for Cateract. By B. Ivy Jeffries, M.D.

A Series of American Clinical Lectures. Edited by E. C. Leguin, M.D. Vol. 1, No. 1.

On Diseases of the Hip-Joint. By Louis A. Sayre, M.D.

Dysmenorrhœa. By. Jno. M. Johnson, M.D., Atlanta, Ga.

Migrants and Sailors considered in their Relation to the Public Health. By Jno. M. Woodworth, M.D., and Herber Smith, M.D.

On Reflex Irritations throughout the Genito-Urinary Tract, resulting from contraction of the urethra at or near the meatus urinarius, congenital or acquired. By Fessenden N. Otis, M.D.

